# Data from a proteomic comparative analysis highlight differential adaptation of *Lactobacillus delbrueckii* subsp. *bulgaricus* to cow milk versus to soy milk environments

**DOI:** 10.1016/j.dib.2022.108653

**Published:** 2022-10-04

**Authors:** Gwénaël Jan, Florian Tarnaud, Fillipe Luiz Rosa do Carmo, Nassima Illikoud, Fanny Canon, Julien Jardin, Valérie Briard-Bion, Fanny Guyomarc'h, Valérie Gagnaire

**Affiliations:** STLO, INRAE, Institut Agro, Rennes F-35000, France

**Keywords:** Yogurt, Starters, Soy milk, Cow milk, Proteomic, Mass spectrometry, Probiotic, Stress

## Abstract

The article presents a proteomic dataset generated by a comparative analysis, using gel-free nanoLC-MS/MS, of the cellular proteome of *Lactobacillus delbrueckii* subsp. *bulgaricus*, a yogurt starter, when cultivated in soy milk versus in cow milk. The CIRM-BIA1592 strain was cultivated in the aqueous phase of soy milk, or of cow milk. Whole-cell proteins were extracted, trypsinolyzed and analyzed by nano LC-MS/MS, prior to identification and to classification by function using the X!Tandem pipeline software and the proteomic data from NCBI.nlm.nigh.gov. Quantification of the proteins was moreover performed to evidence changes in their expression, depending on the culture medium. Data are available via ProteomeXchange with the identifier PXD033905 (http://www.proteomexchange.org/). This article is related to the research article entitled “The stressing life of *Lactobacillus delbrueckii* subsp. *bulgaricus* in soy milk”, by G.Jan et al. in Food Microbiology, 2022. This proteomic differential analysis indeed revealed major modulation of the stress proteome, with many stress proteins upregulated in the soy environment.

**Specifications Table**

**Table:** 

Subject	Food Science: Food Microbiology
Specific subject area	food microbiology, dairy starter, yogurt starter, probiotic, stress adaptation
Type of data	Mass spectrometry data
How the data were acquired	Mass spectrometry. Whole cell protein extracts were subjected to trypsinolysis prior to nano LC-MS/MS analysis.
Data format	Raw, Analyzed, and Filtered
Description of data collection	*Lactobacillus delbrueckii* subsp. *bulgaricus*, strain CIRM-BIA1592, was cultivated either in soy milk, or in cow milk, environment. Whole-cell protein extracts were extracted, trypsinolized, and analyzed by nanoLC-MS/MS in a gel-free strategy. Each peptide identified by tandem mass spectrometry was quantified using the free MassChroQ software before data treatment and statistical analysis under R software.
Data source location	The strain of *Lactobacillus delbrueckii* subsp. *bulgaricus* was provided by the CIRM-BIA (https://collection-cirmbia.fr/), international center for microbial resources, located in the STLO research unit (https://www6.rennes.inrae.fr/stlo). The work was performed at STLO, a research unit of: Institution: INRAE & Institut Agro City: Rennes Country: France
Data accessibility	Data are available via ProteomeXchange with the identifier PXD033905. (http://www.proteomexchange.org/). Repository name: PRIDE (Proteomics Identifications Database) Data identification number: PXD033905 Direct URL to data: https://www.ebi.ac.uk/pride/archive/projects/PXD033905/
Related research article	Jan G, Tarnaud F, Rosa do Carmo FL, Illikoud N, Canon F, Jardin J, Briard-Bion V, Guyomarc'h F, Gagnaire V. 2022. The stressing life of *Lactobacillus delbrueckii* subsp. *bulgaricus* in soy milk. Food Microbiol 106:104042. https://doi.org/10.1016/j.fm.2022.104042

## Value of the Data

• These data constitute an important step in understanding the molecular mechanisms responsible for the adaptation of yogurt starters to the soy environment.
• These data should be considered in the development of innovative fermented soy products.
• These data evidence that the yogurt starter *L. bulgaricus* is stressed when cultivated in soymilk, compared to milk.

## 1 Data Description

This article presents a dataset generated during a comparison of the cellular proteome of the yogurt starter *Lactobacillus delbrueckii* subsp. *bulgaricus* cultivated in (1) cow milk environment and (2) soy milk environment. The list of all the cellular proteins that were identified in this study is provided in a table entitled “protein_quantification_results.csv” available via ProteomeXchange with the identifier PXD033905. Project Name: Data from a proteomic comparative analysis highlight differential adaptation of *Lactobacillus delbrueckii* subsp. bulgaricus to cow milk versus to soy milk environments. Project accession: PXD033905.

The abundance ratios of the differentially expressed proteins, displaying a significantly different abundance when bacteria were cultivated in soy versus in cow milk environment, classified in different COG categories, are published in the following article: [1]. The stressing life of *Lactobacillus delbrueckii* subsp. *bulgaricus* in soy milk. Food Microbiol 106:104042. https://doi.org/10.1016/j.fm.2022.104042.

For each biological and each technical replicate, a raw data file is presented in the deposited dataset as mentioned above.

This article presents data from the research article entitled “The stressing life of *Lactobacillus delbrueckii* subsp. *bulgaricus* in soy milk”. The cellular proteome, according to the growth medium, reveals major induction of stress proteins, in the soy environment.

## 2 Experimental Design, Materials and Methods

### 2.1 Strain and Pre-Cultures

De Man, Rogosa, Sharpe (MRS) medium [2] was used to grow liquid precultures of *Lactobacillus delbrueckii* subsp. *bulgaricus* CIRM-BIA1592 at 42 °C without agitation. This strain was provided by CIRM-BIA, which is an International Centre for Microbial Resources dedicated to bacteria of food interest called CIRM-BIA, (INRAE Rennes, France, https://collection-cirmbia.fr/). These precultures were used to inoculate cultures, either in MRS, or in ultrafiltrate media, as described below.

### 2.2 Culture in Cow Milk or Soy Milk Ultrafiltrate

Raw cow milk was skimmed and then ultrafiltrated on the INRAE STLO dairy platform as described previously [3,4]. In that aim, we used a filtration pilot, containing a ceramic membrane. This membrane has a molecular weight cut-off point of 8 kDa. This ultrafiltration concentrates dairy proteins in the retentate and generates a permeate, MUF (milk ultrafiltrate), which constitutes the aqueous phase of cow milk. The overall composition of MUF was as follows: carbohydrate 5%; non-protein nitrogen 0.28%, minerals 0.75% and dry matter 6.14%. As an additional nitrogen source, 5 g.L^−1^ food grade casein hydrolysate (Casein Peptone Plus, Organotechnie, La Courneuve, France) was added. After adjustment of pH to 7.0 using NaOH, the MUF medium was sterilized using 0.2 µm filters (Nalgene, Roskilde, Denmark) prior to storage at 4 °C.

Soy milk ultrafiltrate (SUF) is a by-product of industrial concentration of soy proteins during the manufacture of soymilk. It was provided by a local company, Sojasun Technologies Triballat Noyal (Noyal-sur-Vilaine, France) and prepared according to patent N° EP 1 983 844 B1 [5]. This SUF is commercially available under the designation BASOSOY (Triballat ingredients Triballat, Noyal-sur-Vilaine, France). The overall composition of SUF was as follows: carbohydrates 2.5%, protein 0.55%, non-protein nitrogen 0.20%, mineral 1%, dry matter 5%. As an additional nitrogen source, 5 g.L^−1^ food grade soy hydrolysate (Bacto Soytone, BD Bioscience) was added. After adjustment of pH to 7.0 using NaOH, the SUF medium was streilized by autoclaving (110 °C, 10 min) prior to sterile centrifugation (28,000 × g, 30 min) and filtration in order to remove insoluble compounds. It was then sterilized using 0.2 µm filters (Nalgene, Roskilde, Denmark) prior to storage at 4 °C.

In this work, 4 independent cultures of *Lactobacillus delbrueckii* subsp. *bulgaricus* CIRM-BIA1592 were prepared at 42 °C without agitation in MUF. In addition, 4 independent cultures of *Lactobacillus delbrueckii* subsp. *bulgaricus* CIRM-BIA1592 were prepared at 42 °C without agitation in SUF. Cultures were incubated until the end of growth and of acidification, i.e. during 16 h. These 8 cultures were then subjected to whole-cell label-free proteomic analysis as described below. For each culture (4 biological replicates), 3 mass spectrometry analyses (3 technical replicates) were performed.

### 2.3 Label-Free Proteomics

Label-free proteomics was performed as described in details in the corresponding research article [1] and adapted from a previous study [6]. Briefly, lactobacilli were grown either in milk or soy ultrafiltrate and then lysed in lysis solution (50 mM Tris-HCl [pH 7.5], 0.3% SDS, 200 mM dithiothreitol (DTT), 0.4 mM phenyl methyl sulfonyl fluoride, PMSF). Protein extracts were cleaned and quantified using the 2-D Clean-Up kit (GE Healthcare) and the 2-D Quant Kit, respectively. Whole-cell tryptic digestion was done using Sequencing Grade Modified Trypsin (Promega, Madison, USA) as described previously [7] prior to addition of spectrophotometric-grade trifluoroacetic acid (TFA) (Sigma-Aldrich, USA).

Nano-LC-MS/MS was done as previously described [7] using a nano RSLC Dionex U3000 system fitted to a Q-Exactive mass spectrometer (Thermo Scientific, San Jose, USA). Spectra of eluted peptides were recorded in full MS mode, selected between 250–2000 m/z, with a resolution of 70,000 at m/z 200.

Identification of proteins was as previously described [7], peptides being identified using the X!Tandem pipeline software (Langella et al., 2017), querying the proteome of *L. delbrueckii* subsp. *bulgaricus* (proteome UP000001259 downloaded from uniprot.org).

Protein quantification was as described [7], each peptide being quantified using the MassChroQ software prior to data treatment and statistical analysis under the R software (R 3.2.2, Project for statistical computing) using the specific R package called 'MassChroqR'. Variance analysis was performed on proteins with a minimum peak ratio of 1.5 for peak counting analysis. We considered significantly different proteins with an adjusted *p*-value < 0.05.

Proteins with a significant (*p* < 0.05, ANOVA) change in expression of ≥ 2-fold (log2 ratio ≥ 1.5) were considered differentially expressed. We generated a volcano plot to illustrate differentially expressed proteins in *L. delbrueckii* subsp. *bulgaricus*. Functional annotation and Clusters of Orthologous Groups (COGs) were done with eggNOG-mapper v2 web tool (Huerta-Cepas et al., 2017, 2019).

The mass spectrometry proteomics data have been deposited to the ProteomeXchange Consortium via the PRIDE [8] partner repository with the dataset identifier PXD033905.

## Ethics Statements

This manuscript adheres to Ethics in publishing standards (https://www.elsevier.com/journals/data-in-brief/2352-3409/guide-for-authors).

## CRediT authorship contribution statement

**Gwénaël Jan:** Writing – review & editing, Visualization, Methodology, Supervision. **Florian Tarnaud:** Writing – review & editing, Methodology. **Fillipe Luiz Rosa do Carmo:** Writing – review & editing, Data curation. **Nassima Illikoud:** Writing – review & editing, Data curation. **Fanny Canon:** Writing – review & editing, Methodology. **Julien Jardin:** Writing – review & editing, Methodology. **Valérie Briard-Bion:** Writing – review & editing, Methodology. **Fanny Guyomarc'h:** Writing – review & editing, Methodology. **Valérie Gagnaire:** Writing – review & editing, Visualization, Methodology, Supervision.

## Declaration of Competing Interest

The authors declare that they have no known competing financial interests or personal relationships that could have appeared to influence the work reported in this paper.

## Data Availability

Data from a proteomic comparative analysis highlight differential adaptation of Lactobacillus delbrueckii subsp. bulgaricus to cow milk versus to soy milk environments (Original data) (PRIDE).
